# Aggressive behaviour among drug-using women from Cape Town, South Africa: ethnicity, heavy alcohol use, methamphetamine and intimate partner violence

**DOI:** 10.1186/s12905-017-0447-2

**Published:** 2017-09-30

**Authors:** Tara Carney, Bronwyn Myers, Tracy L. Kline, Kim Johnson, Wendee M. Wechsberg

**Affiliations:** 1 Alcohol, Tobacco and Other Drug Research Unit, South African Medical Research Council, PO Box 19070, Tygerberg, 7505 South Africa; 2 Department of Psychiatry and Mental Health, University of Cape Town, Cape Town, South Africa; 3 Substance Use Gender, and Applied Research Program, Research Triangle Park, RTI, 3040 Cornwallis Road, Chapel Hill, NC 27709 USA; 4 RTI Gender Global Gender Center, Research Triangle Park, 3040 Cornwallis Road, Chapel Hill, NC 27709 USA; 5 Health, Policy and Administration, Gillings School of Global Public Health, The University of North Carolina, Chapel Hill, NC USA; 6 Psychology in the Public Interest, North Carolina State University, Raleigh, NC USA; 7 Psychiatry and Behavioral Sciences, School of Medicine, Duke University, Durham, NC USA

**Keywords:** Substance use, Aggression, Violence, Women, South Africa

## Abstract

**Background:**

Women have generally been found to be the victims of violence, but scant attention has been paid to the characteristics of women who perpetrate aggression and violence. In South Africa, violence is a prevalent societal issue, especially in the Western Cape.

**Method:**

This study aimed at identifying factors that were associated with aggression among a sample of 720 substance-using women. We conducted multivariate logistic regression to identify factors that are significantly associated with these behaviours.

**Results:**

Ethnicity (Wald Χ^2^ = 17.07(2), *p* < 0.01) and heavy drinking (Wald Χ^2^ = 6.60 (2), *p* = 0.01) were significantly related to verbal aggression, methamphetamine use was significantly related to physical (Wald Χ^2^ = 2.73 (2), p = 0.01) and weapon aggression (Wald Χ^2^ = 7.94 (2), *p* < 0.01) and intimate partner violence was significantly related to verbal (Wald Χ^2^ = 12.43 (2), p < 0.01) and physical aggression (Wald Χ^2^ = 25.92 (2), p < 0.01).

**Conclusions:**

The findings show high levels of aggression among this sample, and highlight the need for interventions that address methamphetamine, heavy drinking and intimate partner violence among vulnerable substance-using women.

## Background

South Africa is a country that is faced with the dual challenges of substance use and violence, with violence being a major contributing factor to death and years lost due to disability [1]. In this country, the Western Cape Province has particularly high levels of substance use disorders and violence perpetration [2, 3]. For example, homicide is a major cause of death in-South Africa, was recorded at 31 per 100, 000 in 2013 in comparison to 6.2 per 100, 000 worldwide in a large global study. In Cape Town, the most recent figures from 2013 are even higher at 59.9% per 100, 000 deaths [4].

Previous research suggests that substance use is a risk factor for violence and crime perpetration. A number of South African studies have found that intentional injuries from interpersonal violence are often linked to alcohol use [4–6], and a study found associations [7] between substance use and the perpetration of violent crimes. Studies have also documented associations between substance use and the perpetration of intimate partner violence [8, 9].

Earlier studies generally focused on men being the perpetrators of violent acts and studied women as the victims. There is a high prevalence of violence against women in some communities in the Western Cape. including among substance-using women whom studies have shown are especially vulnerable to interpersonal violence [10, 11]. While traditionally men who engage in this type of violence have often witnessed it in their childhood and therefore hold traditional views on gender-norms, such gender-disparities in violence perpetration are likely to shift as traditional gender roles in South Africa evolve [12, 13]. This seems to be the case in high-income countries where increasing rates of violence perpetration by women has been documented. For example, studies have shown that women are becoming increasing verbally aggressive [14] and violent [15] in relationships, in response to interpersonal violence from their male partner [16]. In studies conducted in these high-income countries, substance-using women especially have been found to engage in both physical and verbal aggression [17, 18], regardless of reporting any kind of interpersonal violence. Furthermore, there is emerging evidence that being involved in gang activity is associated with higher levels of substance use and aggressive behavior. For example, a recent study found that young women who self-identify as gang members are more likely to use alcohol and other drugs such as marijuana and report engaging in physically violent behaviours, such as fighting with weapons [19].

While substance-using women from the Western Cape may perpetrate aggressive and violent acts, research on this is lacking. In this region, there has been little research on women as perpetrators of violence. An exception has been a limited number of exploratory studies which found that young substance-using women engaged in physical fights with each other [20, 21]. However, this earlier work was largely qualitative and did not examine potential age differences in aggressive behaviour among women; despite evidence that suggests that emerging adulthood (18–25 years of age) is a high risk period for engaging in high risk behaviours such as substance use and aggressive behaviour [22]. There is also a dearth of existing local research on which demographics factors may predispose substance-using women to being aggressive. This paper aims to address this gap through describing aggressive behaviour among substance-using women in Cape Town, South Africa and exploring potential factors associated with verbal, physical and weapon-using aggression among substance-using women.

## Method

### Sample characteristics

The sample consisted of 720 women from disadvantaged communities in Cape Town who were recruited into a randomised controlled trial of a HIV risk reduction intervention for substance-using women between September 2008 and September 2010. Further details of this study have been reported elsewhere [23]. For this paper, we report only on the baseline characteristics of the sample.

To be included in the study, participants had to be women between 18 and 33 years old, live in one of 15 impoverished peri-urban communities, use at least two types of drugs (including alcohol) at least once a week for the past 3 months, be sexually active with a male partner in the past month, provide informed consent to participate in the study and not have participated in the pilot study.

### Research and sampling procedure

We developed a proportional sampling plan to ensure that recruitment was balanced across the broad range of disadvantaged communities in and around Cape Town. Population estimates were used to calculate representative proportions and determine sample sizes within each community. Trained outreach workers used standard street outreach techniques. Outreach workers visited each community and marketed the study with flyers. Marketing posters were placed in local hangout spots, and outreach workers also spoke to women who were possibly eligible. They also handed out marketing pamphlets to women who were possibly eligible in local hangout spots such as bars, shebeens (local taverns), petrol stations and parks. After obtaining verbal permission to administer a brief screening instrument to assess eligibility, they provided eligible and interested women with an appointment for an intake interview and arrangements were made to transport them to our project site for the interview. At this appointment, women were rescreened and asked to provide informed consent to complete a baseline computer-assisted personal interview (which took approximately 90 min) and biological testing for drug use, pregnancy and HIV. Participants were provided with refreshments and a grocery voucher valued at ZAR 40 (USD 3) for their time. Ethics approval for the study was granted by the Institutional Review Boards of RTI International and Stellenbosch University’s Health Research Committee (Trial Registration Number: NCT00729391).

### Measures

The Revised Risk Behaviour Assessment (RRBA) [24] was modified for use in South Africa and used to collect self-report information on socio-demographic characteristics such as their current age, ethnicity, income (individual monthly income from any source), employment and years of education. The RRBA also measures drug and alcohol use history, interpersonal violence and HIV risk behaviours. For the current study, the outcome variable of interest was aggressive behaviours. Participants were asked if they had a main sexual partner, namely a boyfriend or husband, who they could either live with or separately from. They were also asked if they experienced any violence by this partner in the past 6 months. In terms of alcohol use, participants were asked about the number of drinks they consumed on a typical day when drinking, with heavy drinking being defines as seven or more drinks during this occasion. Average days used in the past month were the measures for methamphetamine and cannabis use.

A modified version of the revised conflict tactics scale (CTS2) was used to measure aggressive behaviour. The CTS2 has been found to have acceptable psychometric properties, with a reliability of 0.89 for items included in the scale [25]. It has also been used in previous studies with women [26]. It consists of 10 items that measure three types of aggression: 1) verbal (insulting, swearing at, threatening); 2) physical (including kicking, hitting, biting and throwing objects) and 3) weapon (gun or knife, threatening with gun or knife) aggression in the previous 3 months. All responses to these items are dichotomous (yes/no). Any affirmative responses were scored as indicative of engagement in that type of aggression.

### Data analyses

To explore relationships between verbal, weapon and physical aggression among women that participated in the study, we first conducted bivariate analysis (chi-square tests of assocation for categorical variables and t-tests for continuous variables). We retained demographic variables attaining a significance value of 0.05 or less as potential factors to be controlled in multiple logistic regression models.Three multivariate logistic regression analyses were performed to examine the impact of age, frequency of drinking, cannabis use and methamphetamine use in the past 30 days, and frequency of intimate partner violence in the past 6 months on verbal, physical and weapon aggression in the past 90 days, respectively while adjusting for the potentially confounding effects of the included demographic factors. The SPSS statistical software (version 22) was used for all analysis.

## Results

### Sample description

Out of the total sample (*n* = 720), 460 participants (63.89%) reported engaging in verbal aggression, 292 (40.55%) reported engaging in physical aggression and 57 (7.92%) reported aggression with a weapon in the past 3 months (see Table 1). Young women (18–25 years old) were more likely to have engaged in verbal aggression (X^2^ = 5.28, df = 2, *p* = 0.02), physical aggression (X^2^ = 3.70, df = 2, *p* = 0.05) and weapon aggression (X^2^ = 8.76, df = 2, *p* = 0.03) than older women. The majority of women who reported engaging in verbal (X^2^ = 87.57, df = 2, *p* < 0.01) and physical (X^2^ = 56.51, df = 2, p < 0.01) aggression self-identified as “Coloured” (of mixed race ancestry).

**Table 1:** Factors associated with types of aggression among substance-using women (*n* = 720)

Measure	Verbal Aggression (*n* = 460)	Test Statistic [t(*df*) or χ^2^(*df*)]	p	Physical Aggression (*n* = 292)	Test Statistic [t(*df*) or χ^2^(*df*)]	p	Weapon Aggression (*n* = 57)	Test Statistic [t(*df*) or χ^2^(*df*)]	p
Demographics									
Age (Emerging adult*)	315 (68.5%)	5.28 (2)	0.02*	197 (67.5%)	3.70 (2)	0.05*	31 (54.4%)	8.76 (2)	0.03*
Ethnicity (“Coloured”)	313 (68.0%)	87.57 (2)	<0.01*	210 (71.9%)	56.51 (2)	<0.01*	40 (70.2%)	5.79 (2)	0.02
Average income in past month (M, SD)	306.39 (458.50)	2.93 (718)	<0.01*	366.61 (501.43)	4.61 (718)	<0.01*	312.63 (396.46)	0.75 (718)	0.45
High school education Incomplete (Yes)	414 (90.0%)	1.52 (2)	0.27	266 (91.1%)	2.42 (2)	0.12	52 (91.2%)	0.34 (2)	0.56
Unemployed (Yes)	422 (91.7%)	4.28 (2)	0.04*	268 (91.8%)	1.71 (2)	0.19	52 (91.2%)	0.10 (2)	0.75
Substance Use									
Cannabis Use (M, SD)	14.47 (12.05)	1.61 (562)	0.12	14.15 (11.98)	1.57 (668)	0.16	15.83 (12.38)	0.52 (668)	0.60
Methamphetamine Use (M, SD)	14.35 (9.67)	1.98 (495)	0.05*	15.18 (9.96)	3.09 (495)	<0.01*	18.49 (10.25)	3.50 (495)	<0.01*
Heavy Drinking	208 (45.1%)	35.1 (2)	<0.01*	147 (55.1%)	35.3 (2)	<0.01*	28 (49.1%)	3.2 (2)	0.08
*Main Sexual Partner Violence* (M, SD)	8.84 (10.21)	9.21 (685)	<0.01*	11.13 (10.43)	10.16 (441)	<0.01*	11.89 (11.75)	3.28 (52)	<0.01*

*Emerging adulthood is 18–25 years old

Women with a lower mean monthly income were significantly more likely to have reported engaging in verbal (*t* = 2.93, df = 718, p < 0.01) and physical aggression (*t* = 4.61, df = 718, *p* < 0.01). Compared to employed women, unemployed women were more likely to report engaging in verbal aggression (X^2^ = 4.28, df = 2, *p* = 0.04) but not physical or weapon aggression.

#### Substance use and intimate partner violence

For substance use, women who had a higher average methamphetamine use over the past 30 days were significantly more likely to report all three types of aggression (verbal: *t* = 1.98, df = 495, *p* = 0.05; physical: *t* = 3.09, df = 495, *p* < 0.01; weapon: *t* = 3.50, df = 2, p < 0.01). Heavy drinking was also associated with verbal (X^2^ = 35.10, p < 0.01) and physical aggression (X^2^ = 35.30, p < 0.01). Women who reported engaging in all three kinds of aggression reported a significantly higher average number of days of partner sexual violence in the previous 6 months (*t* = 9.21, df = 685, *p* < 0.01; *t* = 10.16, df = 441, p < 0.01; *t* = 3.28, df = 52, p < 0.01). Education was not entered into any of the three regression models, and nor was employment for physical and weapon regression (*p* > 0.05 in bivariate analysis).

### Multivariate analysis

Ethnicity was significantly associated with verbal aggression (Wald Χ^2^ = 17.07(2), p < 0.01) but not physical or weapon aggression after adjusting for other possible confounding influences in the multiple logistic regression models (Table 2). “Coloured” women were more than three times as likely to report engaging in verbal aggression as black African women (AOR: 3.94; 95% CI: 2.41–6.47). Heavy drinking was also significantly associated with verbal aggression (Wald Χ^2^ = 6.60 (2), *p* = 0.01), where women who reported heavy drinking were almost twice as likely to be verbally aggressive than women who did not report this pattern of drinking (AOR: 1.96; CI: 1.17–3.28). Methamphetamine use was significant associated with physical (Wald Χ^2^ = 2.73 (2), p = 0.01) and weapon aggression (Wald Χ^2^ = 7.94 (2), *p* < 0.01). Women who used methamphetamine had greater odds of engaging in both forms of aggressive behaviour relative to women who used other substances (physical: AOR: 1.03; 95% CI: 2.41–6.47; weapon: AOR: 1.05; 95% CI: 1.02–1.09). Finally, intimate sexual partner violence in the previous 6 months was significantly associated with engaging in verbal (Wald Χ^2^ = 12.43 (2), p < 0.01) and physical aggression (Wald Χ^2^ = 25.92 (2), p < 0.01). After controlling for the influence of other confounding variables, women who reported intimate sexual partner violence had significantly greater odds of being verbally (AOR: 1.06; CI: 1.03–1.10) and physically (AOR: 1.07; CI: 1.04–1.10) aggressive than women without this exposure.

**Table 2:** Logistic regression analysis of variables on aggression-related outcomes

Outcome	Covariate	Wald Test Statistic	AOR*	*p*-value	95% CI
Verbal aggression	Age (Emerging adult) Ethnicity (Coloured) Income Employment Cannabis use Methamphetamine Use Heavy DrinkingMain Sexual Partner Violence	0.50 17.07 0.04 2.76 1.27 1.74 6.6012.43	0.81 3.23 1.00 2.36 1.01 1.02 1.961.06	0.48 <0.01* 0.85 0.10 0.26 0.19 0.01*<0.01*	0.44–1.47 1.85–5.63 1.00–1.00 0.86–6.47 0.99–1.03 0.99–1.04 1.17–3.281.03–1.10
Physical aggression	Age (Emerging adult) Ethnicity (Coloured) Income Cannabis use Methamphetamine Use Heavy Drinking Main Sexual Partner Violence	0.22 1.70 1.90 0.55 2.73 2.73 25.92	0.89 1.42 1.00 1.01 1.03 1.46 1.07	0.64 0.19 0.17 0.46 0.01* 0.10 <0.01*	0.53–1.48 0.84–2.42 1.00–1.00 0.99–1.03 1.01–1.05 0.93–2.29 1.04–1.10
Weapon aggression	Age (Emerging adult) Ethnicity (Coloured) Income Cannabis use Methamphetamine Use Heavy Drinking Main Sexual Partner Violence	0.94 0.59 0.02 2.61 7.94 0.10 3.77	0.67 1.47 1.00 1.03 1.05 1.04 1.04	0.33 0.44 0.96 0.11 <0.01* 0.75 0.05	0.31–1.49 0.55–3.93 1.00–1.00 1.00–1.10 1.02–1.09 0.54–2.35 1.00–1.07

*Adjusted Odds Ratio

## Discussion

This study is one of the first to report on substance-using women’s engagement in various forms of aggressive behaviour, as opposed to being the target of aggression and violence. To the best of our knowledge, there have been limited studies on women’s perpetration of aggression or factors that may influence aggressive behaviour among women. Our findings show that an high proportion of substance-using women engaged in recent acts of aggression. This is a concerning trend as it may increase the risk that violence will be perpetrated against them as indicated by previous studies [8, 14] and may perpetuate cycles of substance use and violence towards women that are often experienced in these communities [27].

The study also sheds some light on which factors that potentially increase substance-using women’s risk of being aggressive. Age was not significantly associated with aggressive behaviour. A possible reason was that the age range of the sample was restricted, with all participants being 33 years old and younger This could possibly explain why the current findings were different to [28] who found that those in emerging adulthood are more likely to be aggressive and violent than older women in established adulthood. Since violence and aggression is so entrenched in the Western Cape region [3, 29], it is possible that women have been exposed to violence for a large proportion of their life, and therefore many women may become aggressive themselves at any age. Previous studies have also shown that there is indeed a long history of violence in South Africa [30].This is especially the case in the Western Cape, where substance-using women have been exposed to an entrenched gang-culture related to violence [21], and there is also evidence that violence against women is inter-generationally transmitted, especially domestic violence [8].

In terms of ethnicity, we found that “Coloured” women had significantly greater odds of self-reporting being verbally aggressive than Black African women. This matches what was found in a previous study with young women, where “Coloured” women reported violence due to substance use and gang violence, with gang initiation involving acts of violence for young women [21]. However, future research is needed as to explain why verbal aggression specifically may be linked to ethnicity.

The study findings also indicated that heavy drinking was significantly associated with verbal aggression after adjusting for confounding variables. This is in keeping with findings from studies with heterosexual couples that found that women are more likely to engage in verbal aggression following alcohol use [17, 30]. The heavy use of alcohol was not related to any physically violent behaviour, as it has been in previous studies with women in specific alcohol-serving settings [18].

Methamphetamine use was significantly related to both physical and weapon aggression. The use of this drug seems to increase the likelihood of women engaging in acts of violence. This finding echoes previous studies conducted in the Western Cape that included women [31, 32], where methamphetamine has been found to be related to the perpetration of aggressive behaviour. Recent research has shown that users’ ability to control or inhibit their aggressive impulses are affected by methamphetamine [33], which could account for heightened aggression and violent behaviour among people who use methamphetamine.

These findings suggest that in order to reduce the perpetration of violence among substance-using women, interventions that focus on the reduction of methamphetamine and heavy alcohol consumption are needed. While methamphetamine use has been found to be prevalent in previous studies with substance-using women in disadvantaged communities [34, 35], treatment for methamphetamine use is still not always accessible for women from these communities, despite high levels of treatment readiness [36, 37]. Providing services that reach such vulnerable substance-using women is therefore key. These services should not only address the substance use, but should also teach women conflict resolution strategies that equip them to manage conflictual situations and limit frustration that may lead to verbal or physical aggression. This is not only important for reducing acts of aggression but also for preventing women from becoming victims of violence.

Another major finding was that participants who had experienced recent violence by their main sexual partner were significantly more likely to exhibit verbal and physical aggression. This is not altogether surprising, since research has found that substance-using women in the Western Cape are at increased risk of interpersonal violence [10]. It is possible that being the victim of intimate partner violence may result in women becoming aggressive and violent themselves [16] if this trauma is left untreated. This may continue the cycle of violence in relationships and can extend to other individuals that encounter substance-using women. To break this cycle, women need access not only to substance use services, but also to trauma and gender-based violence services. In South Africa, there are many barriers to substance-using women accessing mental health services that include trauma support [27]. A previous study conducted in Cape Town found evidence that women do not have equal opportunities to substance use treatment, with barriers including affordability and accessibility to treatment, and concerns about stigma (39). Trauma-informed substance use services are needed for women to prevent the progression of substance use as well as violent behaviour.

These findings need to be considered in the light of several limitations. First, the measurement of aggression was a secondary outcome in the current study, with the focus being on substance use, interpersonal violence and sexual risk. Therefore, the questions around aggression and violence perpetration did not examine the reasons behind aggressive behavior or the relationship that they had with the person towards whom they were aggressive. More research is needed to better understand the perpetration of aggressive behaviour by substance-using women. Second, the sample was recruited from disadvantaged urban communities in greater Cape Town, and it is not known if these findings are representative of other substance-using women in the province or the country. Third, the study did not include young women under the age of 18 years old, and previous work has indicated that adolescence is a developmental period where high risk behaviours are common, including aggression. This highlights the need to further explore these high risk behaviours among younger substance-using women.

## Conclusion

This article provides evidence for high levels of recent aggressive and violent behaviour among substance-using women from poor communities in Cape Town, South Africa. This perpetration of aggressive behaviour should be addressed as this may place women at an elevated risk for exposure to interpersonal violence and legal consequences. Current findings suggest that women who use methamphetamine, drink heavily and have experienced recent intimate partner violence are especially likely to report being aggressive. Comprehensive interventions are needed that provide treatment for substance use disorders, teach women to resolve conflicts without aggression, and provide trauma services for women who have been exposed to physical or sexual violence. Barriers to mental health services for women will also need to be addressed. As South Africa is a highly violent society, identifying the antecedents of aggression and violent behaviour and addressing these in a systematic way to reduce levels of violence is of critical importance to the public health of the country.

## Abbreviations

AOR: Adjusted Odds Ratio
CI: Confidence Intervals
CTS2: Conflict Tactics Scale 2
HIV: Human immunodeficiency virus
RRBA: Revised risk behavior assessment
RTI: Research Triangle Institute
